# Structural insights into *Plasmodium falciparum* nicotinamide mononucleotide adenylyltransferase: oligomeric assembly

**DOI:** 10.1590/0074-02760180073

**Published:** 2018-07-10

**Authors:** Luis Ernesto Contreras-Rodríguez, Catherin Yizet Marin-Mogollon, Lina Marcela Sánchez-Mejía, María Helena Ramírez-Hernández

**Affiliations:** Universidad Nacional de Colombia, Facultad de Ciencias, Laboratorio de Investigaciones Básicas en Bioquímica, Bogotá, Colombia

**Keywords:** NMNAT, oligomers, Plasmodium falciparum

## Abstract

The biochemical pathways involved in nicotinamide adenine dinucleotide (NAD) biosynthesis converge at the enzymatic step catalysed by nicotinamide mononucleotide adenylyltransferase (NMNAT, EC: 2.7.7.1). The majority of NMNATs are assembled into homo-oligomeric states that comprise 2-6 subunits. Recently, the NMNAT of *Plasmodium falciparum* (PfNMNAT) has been identified as a pharmacological target. The enzymatic characterisation, cellular location, and tertiary structure of the PfNMNAT protein have been reported. Nonetheless, its quaternary structure remains to be explored. The present study describes the oligomeric assembly of the 6 x His-PfNMNAT recombinant protein using immobilised metal affinity chromatography coupled with size exclusion chromatography (SEC) and native protein electrophoresis combined with Ferguson plot graphing. These chromatographic approaches resulted in the elution of an active monomer from the SEC column, whereas the Ferguson plot indicated a dimeric assembly of the 6 x His-PfNMNAT protein.

Nicotinamide adenine dinucleotide (NAD) and its phosphorylated form (NADP) are coenzymes involved in REDOX reactions related to energetic metabolism and oxidative stress defence mechanisms. In addition, these molecules are substrates of different enzymes associated with DNA repair and cellular death programs, calcium mobilisation and signalling, genetic expression and circadian rhythm regulation.^1^ NAD biosynthesis can be carried out from diverse chemical precursors through the *salvage* and *de novo* pathways. These routes merge at the enzymatic step catalysed by nicotinamide mononucleotide adenylyltransferase (NMNAT EC: 2.7.7.1).^2^

Functionally, NMNATs are mononucleotide transferases that synthesise the corresponding dinucleotide from nicotinamide mononucleotide (NMN) or its nicotinic acid derivative (NaMN) and ATP. Even though its existence was first discovered in yeast, there have been multiple studies involving NMNATs from Archaea, Bacteria and Eukarya sources. Some organisms possess multiple isoforms whose subcellular distribution and kinetic parameters are distinct.^3^

Multiple X-ray crystallography studies have been carried out to determine the structure of NMNATs from organisms of all three domains of life. These structural analyses have revealed a conserved architecture characterised by the presence of an α/β domain, termed the Rossmann fold, which comprises the elements associated with substrate binding and catalysis.^4^ Regarding quaternary structure, most NMNATs exhibit homo-oligomeric assemblies composed of 2-6 subunits. NMNATs from Gram-positive bacteria, such as *Bacillus anthracis* and *Staphylococcus aureus*, exhibit dimeric associations^5^ ^,^ ^6^, while the NMNAT from *B. subtilis* and human isoform 3 display tetrameric assemblies.^7^ ^,^ ^8^ The most complex organisation has been observed in the NMNAT from *Methanococcus jannaschii* and human isoform 1, which forms hexameric assemblies.^4^ A few NMNATs have been reported to be monomeric, such as the NMNATs from *Escherichia coli* (NadD), *Pseudomonas aeruginosa*, and human isoform 2.^9^ ^-^ ^11^

The vital importance of NMNAT in NAD biosynthesis has established this enzyme as a promising therapeutic target for cancer control^12^, neurodegenerative disorders^13^ and infectious disease treatments.^14^ ^,^ ^15^ In the context of eukaryotic parasites, our research group has identified and characterised NMNATs from *Leishmania braziliensis* (LbNMNAT)^16^, *Giardia lamblia* (GlNMNAT)^17^, and *Trypanosoma cruzi* (TcNMNAT).^18^ Concerning the NMNAT from *Plasmodium falciparum* (PfNMNAT), different functional and structural aspects have been studied.^19^ ^,^ ^20^ However, its quaternary structure has not yet been determined.

This research presents the analysis of the oligomeric state of the 6 x His-PfNMNAT protein through immobilised metal affinity chromatography (IMAC) coupled with size exclusion chromatography (SEC) and native protein electrophoresis (PAGE) combined with Ferguson plot graphing.

Initially, a bioinformatics search in the PlasmoDB database (http://plasmodb.org/plasmo/) permitted the identification of the *pfnmnat* ORF in the genome of the 3D7 strain. The search was performed with the tBLASTn tool using the consensus sequence of multiple previously-characterised NMNATs as query sequences. The identified ORF (PF3D7_1327600) was amplified by polymerase chain reaction (PCR) from 1 mg of *P. falciparum* (FCB2 strain) gDNA, which was extracted with proteinase K and phenol chloroform according to previously described methodologies.^21^ PCR was performed using Platinum Pfx (Invitrogen, Carlsbad, USA)polymerase and the following oligonucleotides: forward 5’-CACCATGCATAAGAATATATGT-3’, reverse 5’-CTAATTAAAATCATATAAGTT-3’. The thermal cycles consisted of an initial denaturation at 94ºC for 2 min, followed by 30 cycles of denaturation at 94ºC for 15 s, annealing at 55ºC for 30 s, and extension at 68ºC for 1 min. Final extension was performed at 68ºC for 7 min. The *pfnmnat fragment* was inserted directly into the pET100/D-Topo (Invitrogen, Carlsbad, USA) expression vector. The identity of the pET100-PfNMNAT recombinant vector was verified by DNA sequencing.

Expression of the corresponding 6 x His-PfNMNAT recombinant protein (~28 kDa) was induced in the *E. coli* Codon Plus (DE3) RIL strain. Luria Bertani medium supplemented with ampicillin (100 mg/mL) and chloramphenicol (50 mg/mL) was used. Protein expression was induced overnight (ON) at 24ºC with IPTG (0.5 mM) when an optical density (OD) of 0.7 at 600 nm was reached. The recombinant protein was fused to an N-terminal 6 x His tag. An IMAC protocol was used employing a nickel nitrilotriacetic acid resin (Ni-NTA) (Qiagen) as follows: induced cells were harvested by centrifugation at 8000 rpm for 15 min at 4ºC, re-suspended in lysis buffer (50 mM NaH_2_PO_4_, 200 mM NaCl, 10 mM imidazole, pH 8.0) (5 mL/g wet weight) supplemented with lysozyme (1 mg/mL) and incubated for 30 min at 4ºC with shaking. Cells were lysed by maceration on liquid nitrogen and centrifuged at 12000 rpm for 20 min at 4ºC. The soluble fraction was incubated for 2 h at 4ºC with 500 mL of Ni-NTA resin pre-equilibrated in lysis buffer. The resin was washed with 15 mL of washing buffer (50 mM NaH_2_PO_4_, 200 mM NaCl, 30 mM imidazole, pH 8.0), and the elution process was carried out with 500 mL of elution buffer (50 mM NaH_2_PO_4_, 200 mM NaCl, 250 mM imidazole, pH 8.0).

**Fig. 1:**
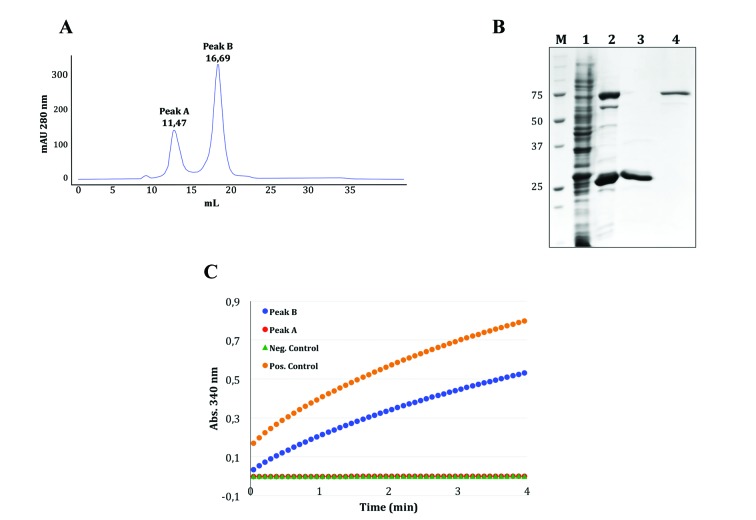
immobilised metal affinity chromatography coupled with size exclusion chromatography (IMAC-SEC) was used to purify an active 6 x His-*Plasmodium falciparum* nicotinamide mononucleotide adenylyltransferase (PfNMNAT) recombinant protein. (A) IMAC eluate 1 was subjected to SEC. The corresponding chromatogram shows two main peaks (A-B). (B) Sodium dodecyl sulfate polyacrylamide gel electrophoresis (SDS-PAGE) 12%T. Coomassie R-250 staining. Lanes: 1. Original soluble fraction; 2. IMAC eluate 1 injected in the SEC column; 3. Peaks B fraction; 4. Peaks A fraction; M. MW (kDa). (C) The enzymatic activity of the 6 x His-PfNMNAT recombinant protein from peak B was verified via coupled enzymatic assays. Negative control: elution buffer. Positive control: 6 x His-*Leishmania braziliensis* nicotinamide mononucleotide adenylyltransferase (LbNMNAT) recombinant protein.^16^

The IMAC 6 x His-PfNMNAT eluates (500 mg) were analysed by SEC in a Superdex 200 10/300 GL column (GE Healthcare Life Sciences) pre-equilibrated in equilibrium buffer (50 mM NaH_2_PO_4_, 300 mM NaCl, 0.5 mM DTT). SEC was carried out in the Äkta Purifier system (GE Healthcare Life Sciences) at 4ºC with a 0.5 mL/min flow rate. Fractions were monitored by absorbance at 280 nm. A molecular weight (MW) calibration curve (partition coefficient (*K*_*av*_ ) *vs* Log MW) was constructed using standard proteins [lysozyme, carbonic anhydrase, bovine serum albumin (BSA)], and 6 x His-HsNMNAT1.^22^

The SEC chromatogram showed two peaks (Fig. 1A) whose fractions were analysed by sodium dodecyl sulfate polyacrylamide gel electrophoresis (SDS-PAGE) and coupled enzymatic assays. SDS-PAGE revealed high MW proteins in peak A, while the 6 x His-PfNMNAT recombinant protein was eluted in peak B (Fig. 1B).

Enzymatic assays were performed with this sample, which showed an increase in absorbance at 340 nm (Fig. 1C). The enzymatic assays were achieved as previously described for other NMNATs.^23^ Initially, the following reaction mix was prepared: 40 mM ethanol, 25 mM HEPES/KOH pH 7.4, 10 mM MgCl_2_, 1.25 mM ATP (Sigma, St. Louis, USA), 1.25 mM NMN (Sigma), and 2 U ADH (Sigma). The mixture was aliquoted into 96-well plates and the reaction was initiated by adding 2.5 mg of the recombinant protein. The reactions were incubated at 37ºC for 4 min. NADH synthesis was monitored at 340 nm in a GENios microplate reader (Teçan, Salzburg, Austria).

The enzymatic activity of the 6 x His-PfNMNAT recombinant protein was confirmed via direct enzymatic assays that measured NAD synthesis from NMN and ATP by reverse phase high-performance liquid chromatography (RP-HPLC) (Fig. 2). These assays were performed using the reaction mixture described above without ethanol and ADH at 37ºC for 10 min. The assays were stopped and neutralised with 1.2 M HClO_4_ and 1 M K_2_CO_3_, respectively.^24^ The reactions were analysed by RP-HPLC in an Agilent 1200 Series instrument with a C18 column (25 cm long x 4.6 mm inner diameter, 5 mm particle size, Phenomenex). A step gradient with buffers A (0.1 mM KH_2_PO_4_, pH 6.0) and B (0.1 mM KH_2_PO_4_, pH 6.0, 20% v/v methanol) was applied as follows: 7 min buffer A (100%), 2 min buffer B (30%), 4 min buffer B (60%), 2 min buffer B (30%), and 2 min buffer A (100%). The analysis was performed at room temperature with a 1.5 mL/min flow rate and a 20 mL injection volume per sample. Analytes were detected at 254 nm.

**Fig. 2:**
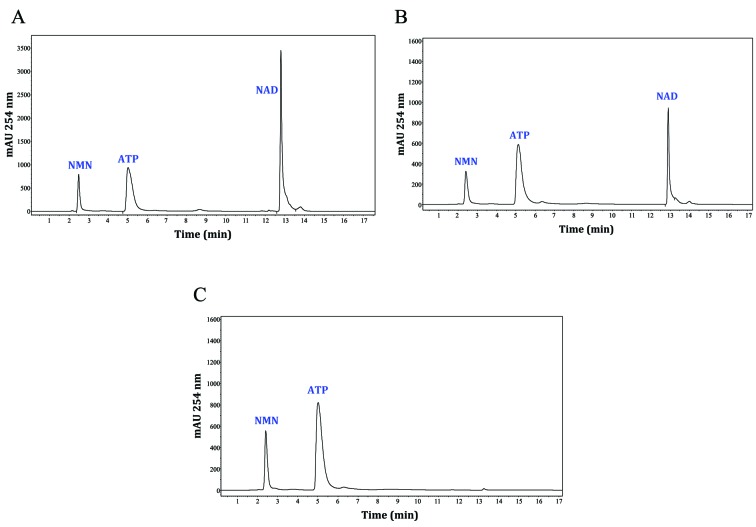
the purified 6 x His-*Plasmodium falciparum* nicotinamide mononucleotide adenylyltransferase (PfNMNAT) recombinant protein was catalytically active. The enzymatic activity of the purified protein was corroborated by direct enzymatic assays and reverse phase high-performance liquid chromatography (RP-HPLC). (A) Standard analytes. (B) Analytes from the 6 x His-PfNMNAT reaction. (C) Negative control reaction (without enzyme).

To study the quaternary structure of the 6 x His-PfNMNAT recombinant protein, the putative oligomeric assembly of the 6 x His-PfNMNAT recombinant protein which eluted in peak B (Fig. 1B) was inferred from its MW. To determine its MW, an MW calibration curve was constructed by injecting a mixture of standard proteins onto the SEC column. From the corresponding chromatogram (Fig. 3), the partition coefficient (*K*_*av*_ ) for each standard was determined (Table), and the calibration curve was generated (Fig. 4). The elution volume of the 6 x His-PfNMNAT recombinant protein eluted in peak B (16.69 mL) and was interpolated from the calibration curve to obtain a value of 36.05 kDa. This value is close to the theoretical MW of the monomeric recombinant protein, which is ~28 kDa. Recently, monomers of the PfNMNAT protein were observed via X-ray crystallography.^20^

**TABLE Partition:** coefficients (*K*_*av*_ ) of the protein standards analysed by size exclusion chromatography (SEC)

Standard	Molecular weight (MW) (Da)	Log MW	Elution volume (V_e_) (mL)	*K*_*av*_ = V_e_-V_o_/V_c_-V_o_
1: 6xHis-HsNMNAT1 (4-mer)	132000	5,12	12,27	0,26
2: BSA	66463	4,82	14,75	0,41
3: Carbonic anhydrase	29000	4,46	16,85	0,55
4: Lysozyme	14307	4,16	19,76	0,73
Additional data				
Dextran blue				Void volume (V_o_) = 8,22 mL	
Column: Superdex 200 10/300 GL				Column volume (V_c_) = 24 mL	

**Fig. 3:**
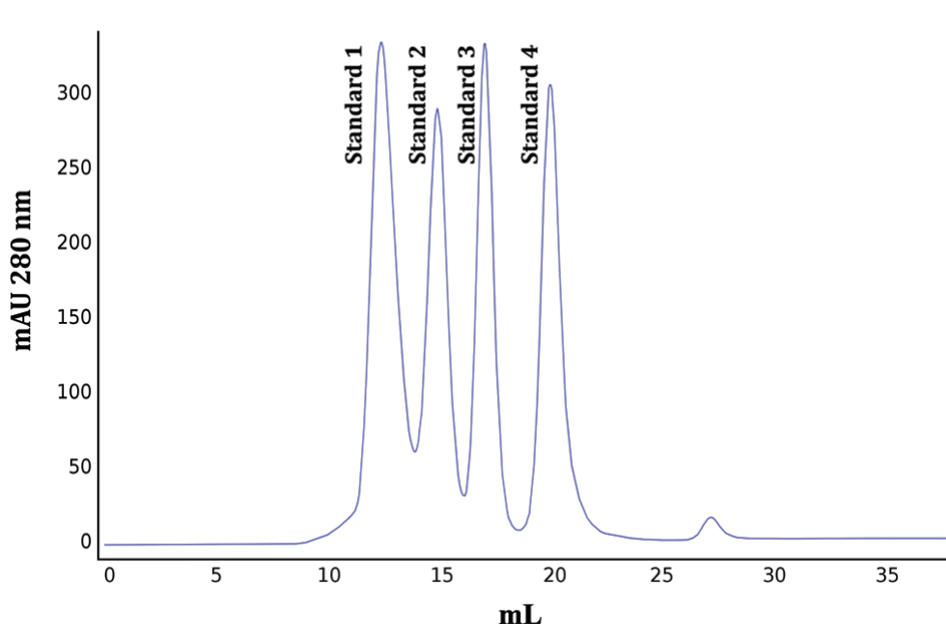
molecular weight (MW) analysis of protein standards by size exclusion chromatography (SEC). The standardised SEC conditions separated the protein standards referred to in Table.

Our analysis of the quaternary structure of the 6 x His-PfNMNAT recombinant protein by IMAC-SEC revealed that the protein exists in a monomeric and active state. This evidence agrees with previous observations for NMNATs from *E. coli* (NadD) and *P. aeruginosa*, whose monomeric states are capable of NAD synthesis.^9^ ^,^ ^10^ Thus, it is reasonable to conclude that the quaternary structure is not a necessary requirement for NMNATs to be active, according to previous observations.^4^ The SEC assay was completed in the absence of any substrate or cofactor. Consequently, it would be interesting to evaluate the effect of these molecules on the oligomeric assembly of the 6 x His-PfNMNAT recombinant protein. Other studies have confirmed certain effects: via X-ray crystallography, dimers and tetramers of the NMNAT from *P. aeruginosa* have been observed in the presence of ATP and NaMN, respectively.^10^ Furthermore, the NMNAT from *B. subtilis* forms a tetramer in the absence of substrates and a dimer in the presence of one of its products (nicotinic acid adenine dinucleotide, NAAD).^7^

**Fig. 4:**
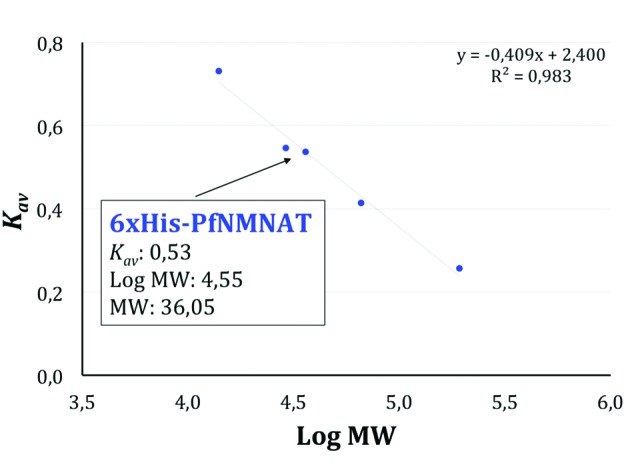
the 6 x His-*Plasmodium falciparum* nicotinamide mononucleotide adenylyltransferase (PfNMNAT) recombinant protein eluted as a monomer from the size exclusion chromatography (SEC) column. Molecular weight (MW) calibration curve. The equation of the curve and the *K*_*av*_ value were used to calculate the MW of the 6 x His-PfNMNAT recombinant protein.

To acquire additional information on the quaternary structure of the 6 x His-PfNMNAT recombinant protein by means of complementary techniques, analysis by PAGE and Ferguson plot graphing was performed. Briefly, the IMAC 6 x His-PfNMNAT eluates (20 mg) were analysed by PAGE, similar to that indicated in Fig. 5A. The following standard proteins (40 mg) were loaded: lactalbumin, trypsin inhibitor, carbonic anhydrase, ovalbumin and BSA. Four parallel gels, each with a specific acrylamide/bis-acrylamide concentration (%T) (6, 8, 10, 12), were run, and the relative mobility (*R*_*f*_ ) was measured for each sample. Retardation coefficients (*K*_*r*_ ) were calculated from the slope of a Log *R*_*f*_ vs %T graph (Fig. 5B), and the Ferguson plot was constructed (-Log *K*_*r*_ *vs* Log MW; Fig. 5C). In parallel, the *K*_*r*_ of the intense band corresponding to the 6 x His-PfNMNAT recombinant protein (Fig. 5A, lane 6) was interpolated in the Ferguson plot. An MW value of 54.5 kDa was obtained. This result is in concordance with a dimeric assembly of the 6 x His-PfNMNAT recombinant protein. Similar results (dimeric assemblies) have been reported for NMNATs from *B. anthracis* and *S. aureus*.^5^ ^,^ ^6^.

**Fig. 5:**
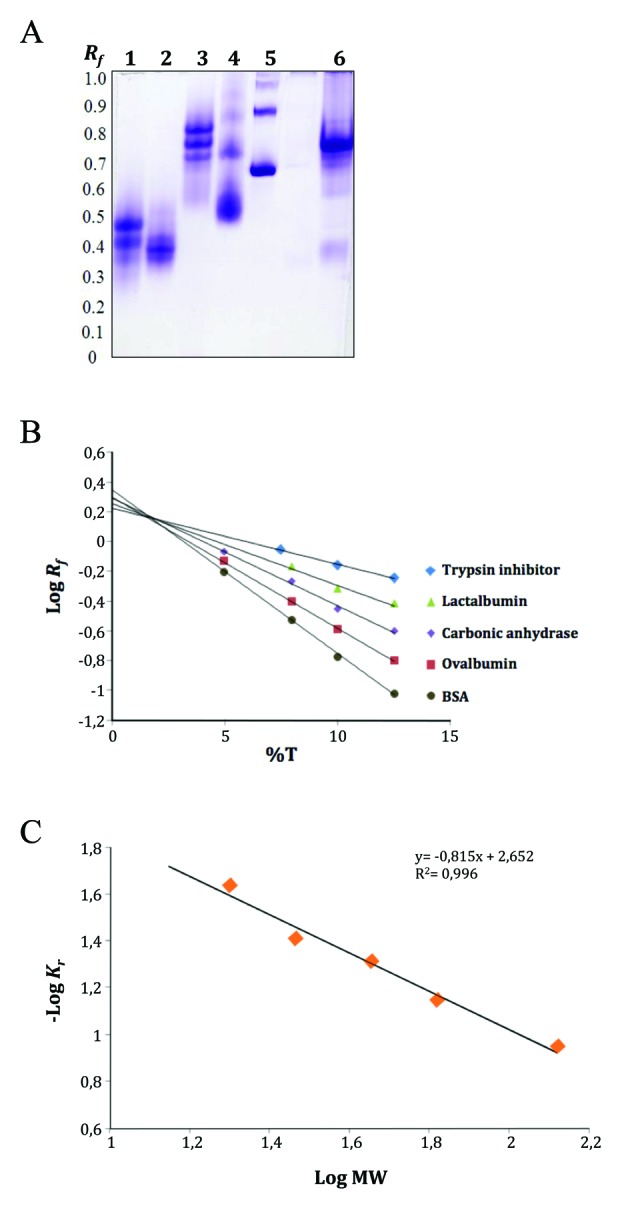
native protein electrophoresis (PAGE) and Ferguson plot analysis of the protein standards and the 6 x His-*Plasmodium falciparum* nicotinamide mononucleotide adenylyltransferase (PfNMNAT) recombinant protein. (A) native protein electrophoresis (PAGE) 10%T. Coomassie R-250 staining. Lanes: 1. Lactalbumin (14 kDa); 2. Trypsin inhibitor (20 kDa); 3. Carbonic anhydrase (29 kDa); 4. Ovalbumin (45 kDa); 5. BSA (66 kDa); 6. 6 x His-PfNMNAT. (B) %T effect on the relative mobility (*R*_*f*_ ) of the standards proteins. (C) Ferguson plot. The -Log of the slopes from figure in panel B (retardation coefficients, *K*_*r*_ ) is plotted as a function of the Log molecular weight (MW) of the corresponding standards.

In contrast to the IMAC-SEC experiment, the Ferguson plot indicated that the 6 x His-PfNMNAT recombinant protein formed a dimeric assembly. In agreement with this result, NMNATs with an observed quaternary structure were characterised by the presence of a structural element implicated in the interaction of subunits.^4^ This element comprises a long loop or a loop and a small β strand that connects the Rossmann fold to the C-terminal domain. Analysis of the recently-resolved PfNMNAT protein structure^20^ revealed that this structural element occurs in the PfNMNAT protein structure (Fig. 6D). Taken together, these observations reinforce our finding and highlight the potential for the PfNMNAT protein to establish oligomeric assemblies.

**Fig. 6:**
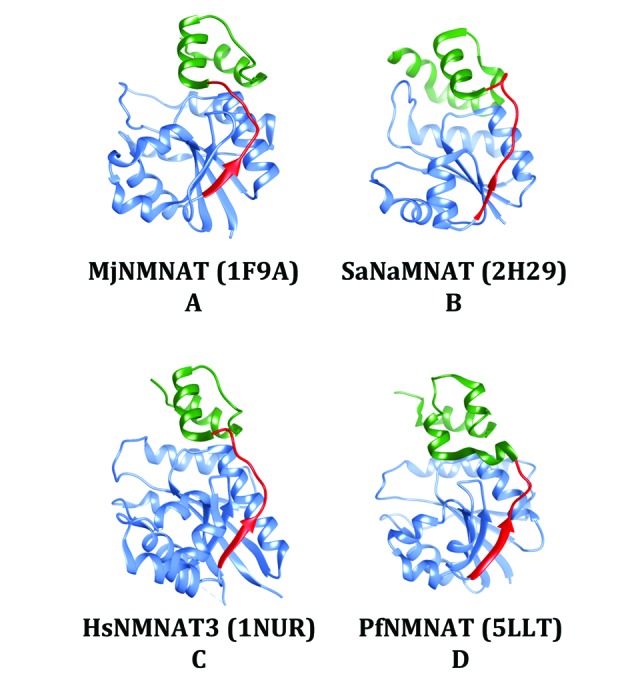
the *Plasmodium falciparum* nicotinamide mononucleotide adenylyltransferase (PfNMNAT) protein possesses the structural elements involved in oligomeric assembly. The quaternary structures of NMNATs exhibit a common structural element (red) between the Rossmann fold (blue) and the C-terminal domain (green). (A-D) NMNAT structures solved by X-ray crystallography. MjNMNAT, SaNaMNAT, HsNMNAT3, PfNMNAT: NMNATs from *Methanococcus jannaschii*, *Staphylococcus aureus*, *Homo sapiens* and *Plasmodium falciparum*, respectively. The image design was adapted from.^4^ Protein Data Bank (PDB) codes are indicated in parentheses. The image was generated with UCSF Chimera.^29^

The differences among the results derived from the IMAC-SEC experiment and the Ferguson plot, could be attributed to the specific properties of these techniques, such as the buffer systems, matrices (Sephadex vs polyacrylamide) and the experimental principles (filtration vs electrophoresis). In accordance with this notion, it has been reported that the endogenous and recombinant HsNMNAT1 protein behaves as a tetramer in SEC assays.^25^ ^,^ ^26^ However, independent X-ray crystallography studies of this protein indicated a hexameric assembly.^27^ ^,^ ^28^ The identification of different oligomeric assemblies by the same NMNAT could be related to regulatory mechanisms, which are activated under certain biological conditions.
