# ERRATUM: Structural insights into *Plasmodium falciparum* nicotinamide mononucleotide adenylyltransferase: oligomeric assembly”

**DOI:** 10.1590/0074-02760180073ER

**Published:** 2018-08-09

In the article “**Structural insights into *Plasmodium falciparum* nicotinamide mononucleotide adenylyltransferase: oligomeric assembly**", DOI number: 10.1590/0074-02760180073, published in *Mem Inst Oswaldo Cruz*, Rio de Janeiro, Vol. 113(9): e180073, 2018, on page 1:

Where it reads:

ORIGINAL ARTICLE

It should read:

SHORT COMMUNICATION

http://dx.doi.org/10.1590/0074-02760180073ER

